# Editorial: Neural oscillations in physiology and neuropsychiatric disorders

**DOI:** 10.3389/fnhum.2022.1016481

**Published:** 2022-08-31

**Authors:** Shozo Tobimatsu

**Affiliations:** Department of Orthoptics, Faculty of Medicine, Fukuoka International University of Health and Welfare, Fukuoka, Japan

**Keywords:** neural oscillations, neuropsychiatric disorders, EEG, magnetoencephalography (MEG), non-invasive brain stimulation (NIBS), time-frequency (TF) analysis

Oscillatory neuronal (electrical) activity in defined frequency ranges supports synchronous interactions between anatomically distinct regions of the human brain during cognitive tasks (Singer, 1999, 2018). From our previous studies (Tobimatsu, 2020a,b), altered neural synchronization plays an important role in distributed cortico-cortical processing. Thus, some neuropsychiatric disorders can be conceptualized as network diseases. Interestingly, the development of non-invasive brain stimulation techniques such as repetitive transcranial magnetic stimulation (rTMS) and transcranial alternating current stimulation (tACS) enables us to manipulate the brain oscillations and brain function in human (Vosskuhl et al., 2018).

Taken together, the goal of this Research Topic is to conceptualize the brain as a self-organizing complex system in which numerous, densely interconnected, but functionally specialized areas cooperate in context- and task-dependent constellations. In the following, a total of nine articles focusing on cognitive abnormalities and underlying oscillatory dysfunctions in animals and humans were published.

Kajita et al. reported “*Heterogeneous GAD65 Expression in Subtypes of GABAergic Neurons Across Layers of the Cerebral Cortex and Hippocampus*.” Neuronal oscillations are modulated by the excitatory-inhibitory balance among the neurons. They found that each GABAergic subtype exhibited a distinct GAD65 expression pattern across layers of the cerebral cortex and hippocampus in colchicine-treated rats. These findings suggest that exploration of the distinct profiles of GAD65 expression among GABAergic subtypes could clarify the roles that GABAergic subtypes play in maintaining the excitatory-inhibitory balance.

Sakamoto et al. reported “*Shape and Rule Information Is Reflected in Different Local Field Potential Frequencies and Different Areas of the Primate Lateral Prefrontal Cortex*.” They analyzed how local field potentials (LFPs) recorded from the monkey lateral prefrontal cortex (LFPC) were modulated by the crucial factors of a shape manipulation task. The transformed shape in the sample period strongly affected the theta and delta waves in the delay period on the ventral side, while the arm-manipulation assignment influenced the gamma components on the dorsal side. Thus, area- and frequency-selective LFP modulations are involved in dynamically recruiting different behavior-relevant information in the LFPC.

Saito et al. reported “*D1 Receptor Mediated Dopaminergic Neurotransmission Facilitates Remote Memory of Contextual Fear Conditioning*.” They studied the role of dopaminergic neurotransmission *via* dopamine D1 receptors (D1Rs) in aversive memory formation in contextual and auditory cued fear conditioning tests using D1R knockdown (KD) mice, in which the expression of D1Rs could be conditionally and reversibly controlled with doxycycline (Dox) treatment. When D1R expression was suppressed using Dox, behavioral experiments revealed impaired contextual fear learning in remote aversion memory following footshock stimulation. Thus, deficiency in D1R-mediated dopaminergic neurotransmission is an important factor in impairing contextual fear memory formation for remote memory.

Okazaki et al. published “*Frequency- and Area-Specific Phase Entrainment of Intrinsic Cortical Oscillations by Repetitive Transcranial Magnetic Stimulation*.” They tested whether spontaneous neural oscillations in different local cortical areas and large-scale networks can be phase-entrained by direct perturbation with distinct frequencies of rTMS in humans. rTMS at 23 Hz over the motor cortex and 11 Hz over the visual cortex induced a prominent and progressive increase in phase-locking factor (PLF) that lasted for a few cycles after the termination of rTMS. Moreover, the local increase in PLF propagated to other cortical areas. These results suggest that distinct cortical areas have area-specific oscillatory frequencies, and the manipulation of oscillations in local areas impacts other areas through the large-scale oscillatory network with the corresponding frequency specificity.

Gordon et al. reported “*Prefrontal Theta-Phase Synchronized Brain Stimulation With Real-Time EEG-Triggered TMS*.” They investigated individual source-space beamforming-based estimation of the prefrontal theta oscillation as a method to target specific phases of the ongoing theta oscillations in the human dorsomedial prefrontal cortex (DMPFC) with real-time EEG-triggered TMS. Using optimized parameters, prefrontal theta-phase synchronized TMS of DMPFC was achieved with an accuracy of ±55°. This method is relevant for brain state-dependent stimulation in human studies of cognition. It will also enable new personalized therapeutic repetitive TMS protocols for more effective treatment of neuropsychiatric disorders.

Ogata et al. published “*After-Effects of Intermittent Theta-Burst Stimulation Are Differentially and Phase-Dependently Suppressed by* α*- and* β*-Frequency Transcranial Alternating Current Stimulation*.” Intermittent theta-burst stimulation (iTBS) using TMS is known to produce excitatory after-effects over the primary motor cortex (M1). They tested their hypothesis that tACS would modulate the after-effects of iTBS depending on the stimulation frequency and phase using motor evoked potentials (MEPs). α-tACS suppressed iTBS effects at the peak phase but not at the trough phase, while β-tACS suppressed the effects at both phases. Thus, although both types of tACS inhibited the facilitatory effects of iTBS, only α-tACS did so in a phase- dependent manner. In conclusion, the action of iTBS is differentially modulated by neuronal oscillations depending on whether α- or β-tACS is applied.

Kobayashi et al. reported “*Exclusion of the Possibility of “False Ripples” From Ripple Band High-Frequency Oscillations Recorded From Scalp Electroencephalogram in Children With Epilepsy*.” Ripple-band epileptic high-frequency oscillations (HFOs) can be recorded by scalp EEG in association with epileptic spikes. But the filtration of steep waveforms such as spikes may cause spurious oscillations or “false ripples.” They have demonstrated that the numerical differentiation of EEG data provides convincing evidence that HFOs were detected in terms of the presence of such unusually fast oscillations over the scalp and the importance of this electrophysiological phenomenon.

Sultana et al. reported “*A Long Time Constant May Endorse Sharp Waves and Spikes Over Sharp Transients in Scalp Electroencephalography: A Comparison of After-Slow Among Different Time Constants Concordant With High-Frequency Activity Analysis*.” They examined whether long time constant (TC) is useful for detecting the after-slow activity of epileptiform discharges (EDs): sharp waves and spikes and for differentiating EDs from sharp transients (Sts). Compared to Sts, high-frequency activity (HFA) was found significantly more with the apical component of EDs. Thus, long TC could be useful for selectively endorsing after-slow of EDs and differentiating EDs from Sts.

Roberts et al. published “*Magnetoencephalography Studies of the Envelope Following Response During Amplitude-Modulated Sweeps: Diminished Phase Synchrony in Autism Spectrum Disorder*.” Auditory steady-state responses (ASSR, driven at 40 Hz) can elicit coherent electrophysiological responses from intact circuitry in adults. Thus, the authors applied amplitude-modulated (AM) sweep stimuli (500 Hz carrier; sweep 10–100 Hz up and down) bilaterally to 40 typically developing and 80 children with autism spectrum disorder (ASD). They found an imbalance of excitatory and inhibitory neurotransmission in MEG and concluded that (1) the AM sweep stimulus provides a mechanism for probing ASSR in an unbiased fashion, during developmental maturation of peak response frequency, (2) peak frequencies vary, in part due to developmental age, and importantly, (3) intra-trial coherence (ITC) at this peak frequency is diminished in ASD, with the degree of ITC disturbance related to clinically assessed language impairment.

In conclusion, we hope that these papers will shed light on the nature of brain oscillations in animals and humans.

## Author contributions

The author confirms being the sole contributor of this work and has approved it for publication.

## Funding

This work was supported in part by Grant-in-Aid for Scientific Research on Innovative Areas MEXT KAKENHI 15H05875.

## Conflict of interest

The author declares that the research was conducted in the absence of any commercial or financial relationships that could be construed as a potential conflict of interest.

## Publisher's note

All claims expressed in this article are solely those of the authors and do not necessarily represent those of their affiliated organizations, or those of the publisher, the editors and the reviewers. Any product that may be evaluated in this article, or claim that may be made by its manufacturer, is not guaranteed or endorsed by the publisher.
